# Assessing the Impact of Socioeconomic Variables on Small Area Variations in Suicide Outcomes in England

**DOI:** 10.3390/ijerph10010158

**Published:** 2012-12-27

**Authors:** Peter Congdon

**Affiliations:** School of Geography, Queen Mary University of London, Mile End Rd, London E1 4NS, UK; E-Mail: p.congdon@qmul.ac.uk; Tel.: +44-20-7-882-8200; Fax: +44-20-7-882-7479.

**Keywords:** suicide, self-harm, deprivation, fragmentation, rurality, spatial, nonlinear

## Abstract

Ecological studies of suicide and self-harm have established the importance of area variables (e.g., deprivation, social fragmentation) in explaining variations in suicide risk. However, there are likely to be unobserved influences on risk, typically spatially clustered, which can be modeled as random effects. Regression impacts may be biased if no account is taken of spatially structured influences on risk. Furthermore a default assumption of linear effects of area variables may also misstate or understate their impact. This paper considers variations in suicide outcomes for small areas across England, and investigates the impact on them of area socio-economic variables, while also investigating potential nonlinearity in their impact and allowing for spatially clustered unobserved factors. The outcomes are self-harm hospitalisations and suicide mortality over 6,781 Middle Level Super Output Areas.

## 1. Introduction

Ecological studies of suicide and self-harm investigate geographical variations in risk and their association with explanatory variables, some of which may be measured and some unobserved. For example, Boyle *et al*. [1] present evidence of contrasting area suicide mortality according to area socioeconomic status, as measured by area deprivation scores. A number of studies of suicide and psychiatric morbidity also establish the role of area household structure and population turnover, as summarized in the so-called social fragmentation index [2]. Urban-rural variation in suicide outcomes is also well documented [3].

Even after accounting for observed influences, there is likely to be remaining variability which typically shows strong spatial clustering [4,5]. Effects of known predictors may be biased if no account is taken of spatially structured residual variation, that is of spatial confounding [6,7,8]. Furthermore regression impacts of predictors (e.g., fragmentation) may be understated or misstated if a default assumption of linearity is adopted when in fact there are nonlinear effects [9,10].

This paper considers small area variations in self-harm (hospital stays) during 2006/2007 to 2010/2011 (five financial years), and in suicide mortality (total and by gender) during 2006–2010. The specific area framework is 6,781 small areas across all of England (Middle Level Super Output Areas), averaging 7,600 in total population. The focus is on establishing whether there is a significant impact on suicide and self-harm across English small areas of deprivation, fragmentation and rurality.

The analysis has as its primary intention to add to the existing literature on area variations in suicide and provide a broad scale perspective on ecological (area level) risk factors for self-harm and suicide. Nevertheless methodological choices are important given the nature of the data (count data, with often small event counts for suicide in particular), the latent nature of the ecological risk factors, the presence of unobserved influences, and also the need to ensure that the relationship between outcome and risk factor is adequately represented (e.g., avoiding linearity by default).

The wide geographic coverage of the analysis contrasts with most previous studies of area suicide variation within the UK, which have been within particular cities or regions. This spatially extensive framework, combined with methodological consideration of spatial structured residual variation and potential nonlinearity, provides a firm base for establishing the effects of area socioeconomic variables on small area suicide outcomes. The following section considers methodological issues (e.g., form of regression), Section 3 considers measurement of the latent area risk factors, Section 4 considers the available data, Section 5 discusses aspects of the methods applied in the case study, and Section 6 describes the results of the regression analysis. Section 7 reviews the evidence obtained in a wider context.

## 2. Methods

A number of techniques are available for spatially adapted regression, namely regression allowing for spatially correlated residuals or other sorts of spatial dependency. As noted by Earnest *et al*. [11], “traditional regression models are not adequate” for analysing spatial data on health outcomes because they fail to account for geographic correlation in the data, with observations from areas close together tending to have similar values. This correlation reflects spatial structure in the covariate risk factors affecting the outcomes, some of which may be unmeasured. Such unobserved risk factors may encompass both area characteristics and the occurrence of suicide clusters of a contagious imitative character [12,13]. The unmeasured risk factors may be modelled by using a random effect with spatial structure in a hierarchical model [11,14]. Here this hierarchical model is estimated using Bayesian methods as implemented in the INLA package within the freeware R package [14,15], though “classical” techniques such as penalized quasi-likelihood can be used instead. A conditional autoregressive scheme is adopted for the spatially structured random effect, whereby a spatial effect for a particular area is normally distributed with its mean given by an average of effect values in neighbouring areas.

Hierarchical models are an extension of generalized linear models, with a mean function comprising a covariate component, and one or more random effects. If there are no measured covariates to model the spatial pattern, then the spatial random effect represents the spatial pattern in the outcomes, whereas otherwise it captures spatial structure in the residuals.

Because the two outcomes are counts, including possibly zero event totals, a Poisson regression model is adopted, so following a widely adopted methodology for modelling area suicides (e.g., [16,17]). Often [18,19] there may be evidence of overdispersion of the counts with respect to the Poisson model as well as spatial patterns, so requiring an additional random effect (a “pure heterogeneity” effect) to model this form of heterogeneity. Overdispersion is a term for variance exceeding the mean, contrary to the Poisson assumption. A heterogeneity random effect may not always be necessary, and the analysis below compares model fit between specifications including both spatial and heterogeneity effects, and specifications with spatial effects only.

Let y_i_ denote an observed total of self-harm events or suicide deaths in the ith small area, i = 1,..,6781, with a Poisson distribution for both outcomes assumed subject to the possible need for a random effect to account for overdispersion. The Poisson mean can be represented as the product E_i_ρ_i_ of an expected number E_i_ of deaths or self-harms and a relative risk ρ_i_ (analogous to an SMR but with national average 1 rather than 100). Expected events E_i_ are obtained by standard demographic methods, namely applying England wide age rates to small area populations. Elevated suicide or self-harm risks in area i corresponds to ρ_i_ in excess of 1.

In a model including linear effects of p covariates X_i_ = (X_i1_,..,X_ip_), spatially structured random effects s_i_, and a normally distributed heterogeneity effect h_i_, a log link regression would take the form of Equation (1):

log(ρ_i_) = α + X_i_β + s_i_ + h_i_ (1)

The linearity assumption here means that a given difference ∆_X_ in X values translates into the same effect on log relative risk (namely β∆_X_) whatever the value of X. However, default linearity assumptions may oversimplify and may be assessed against nonlinear alternatives. For example, the increase in suicide risk (for a particular set difference ∆_X_ in X values) at high X values may be less than the increase in risk at intermediate or low X values. Simple alternatives are polynomial models with quadratic or cubic terms in the independent variable(s), or applying cutpoints to categorize the independent variable, implying regression models with step functions. The latter option discards information [20] while polynomial functions may not provide adequate flexibility [21]. The goal is to provide a more flexible regression function under the weaker assumption that the regression effect is smooth but not necessarily linear [21].

Thus one may assume smoothly varying functions s(X) over the range of predictor values [10], as in Equation (2):

log(ρ_i_) = α + S_1_(X_i1_) + …S_p_(X_ip_) + s_i_ + h_i_ (2)

whereby the effect of a given change in X on log relative risk is of unknown form. The model assumed for S(X) allows for the slope to slowly increase or fall as X varies, so that the effect of X on relative suicide risk depends on the value of X itself. In the R-INLA package, a smoothly changing regression effect can be achieved by random walk schemes, and in the case study of English suicide rates below a first order random walk assumption is adopted. To provide additional evidence for the presence or not of nonlinear regression effects a cubic spline regression approach is also considered. As mentioned by Vittinghoff *et al*. [21] splines provide more flexibility while preserving continuity and smoothness, with changes in slope at knots or cutpoints in the range of the predictor.

## 3. Measuring Risk Factors for Area Suicide Variation

Ecological measures of socioeconomic status (SES) are widely used in studies of spatial health inequality, with summary indices of area SES or social deprivation based on indicators such as unemployment, income levels or property values. A number of studies report that deprivation increases risk for suicide outcomes, both self-harm and completed suicide [22,23], with effects generally found to be stronger on self-harm. Impacts of area SES on ecological suicide variations reflect the role of individual level SES on suicide risk, though area SES effects on suicide combine both effects of area population structure (“compositional” effects), and effects of area per se (*i.e.*, “contextual” effects) [24].

In the present study a summary index of area SES is based on four indicators available for MSOAs, namely (1) Percentage households in poverty (2007–2008) (2) Average Weekly Household Total Income Estimate (2007–2008) (3) Unemployment rate among working ages (2008) (4) Income Support Claimants, % Population (2008). The first, third and fourth variables are intended to represent poverty, which is recognized to be significant both in working households as well as among the economically inactive and welfare dependent [25]. By contrast the second variable is a positive measure of area socio-economic status, and is likely to capture highly affluent MSOAs (and variations in area SES between them) as well as areas with low average incomes. A widening suicide gap between the most and the least deprived areas has been noted in recent studies [13]. The deprivation score used subsequently in the regression analysis (Section 6) is based on principal component analysis of these variables (in STATA), which shows the leading component accounts for 79% of the original variation in the indices.

A possible alternative to the adopted strategy would be to use the UK government’s Index of Multiple Deprivation (or IMD). This was developed at a lower spatial scale (Lower level Super Output Areas, or LSOAs) than the MSOAs used in the present study, and deriving an MSOA score would involve an ad hoc averaging over LSOAs within each MSOA. Another issue concerns the inclusion in the IMD of a health domain introducing potential confounding with the suicide outcomes [26].

A number of studies have considered the impact on suicide outcomes of an index most commonly denoted as social fragmentation [22,27], meaning relatively low levels of community integration linked to high numbers of nonfamily households (e.g., one person households, unmarried adults), and high residential turnover. For example, an index of area fragmentation [28] proposed for small areas in London was based on one person households, renting from private sector landlords, residential turnover, and non-married adults.

Fragmentation is an ecological measure of individual level risk factors for suicide, for example living alone, residential transience and being unmarried [29,30], and may also represent contextual effects, such as adverse impacts of high neighbourhood transience on population mental health [31]. Deprivation and fragmentation are distinct conceptually, since fragmentation primarily measures household and family composition and migrant turnover, and is not intrinsically linked to socioeconomic status [32].

A summary index of social fragmentation is based on four indicators: (1) One person households, as percent of all households (2001 Census) (2) Married couple households with dependent children, % all households (2001 Census) (3) Migrant inflow, % population (2008–2009) (4) Migrant outflow, % population (2008–2009). The indicators are intended to capture different aspects of residential transience and a non-family household structure in certain small areas, as opposed to of familism and residential stability. The choice of indicators differ from those in the 1996 study by Congdon [28] since changes in the UK housing market mean that private sector renting (used as an indicator of fragmentation in that study) is less clearly associated with a transient life style and non-family households. The second indicator is a negative measure of fragmentation, while the others are positive measures. A fragmentation score is obtained from principal component analysis of these variables, which shows the leading component accounts for 77% of the original variation in the indices. The fragmentation scores have a correlation of 0.51 with the deprivation scores.

A number of UK and European studies have found an association between urban-rural residence and suicide outcomes, though the gradient differs between outcome (self-harm as against suicide) and between countries. For example, [33] find excess rural suicide in the US, and a widening of the rural-urban gradient, and some UK studies [34,35,36] also report excess rural suicide. Contextual aspects of the rural economy and healthcare may be relevant to suicide contrasts, such as relatively poor access to psychiatric services, and easier access to lethal suicide methods.

By contrast, [37] consider variation within one English county (Oxfordshire) and report higher rates of self-harm in urban areas, while [38] consider NW England, and report “higher (self-harm) ratios are shown to be localized within urban areas, though in the case of self-harm, Liverpool and central Manchester do not show the typical high ratio levels seen for many health issues linked to deprivation”.

The present study has a more inclusive coverage (across all of England) than earlier studies, and adopts a continuous rurality index (see below). Previous studies often use simple categorization such as a binary urban-rural distinction, or the three fold categorization in [37], whereas categorization of an underlying continuum may have drawbacks such as loss of efficiency [39,40]. A simple categorization may also obscure within city variations such as inner city *vs.* suburban variations.

**Figure 1:**
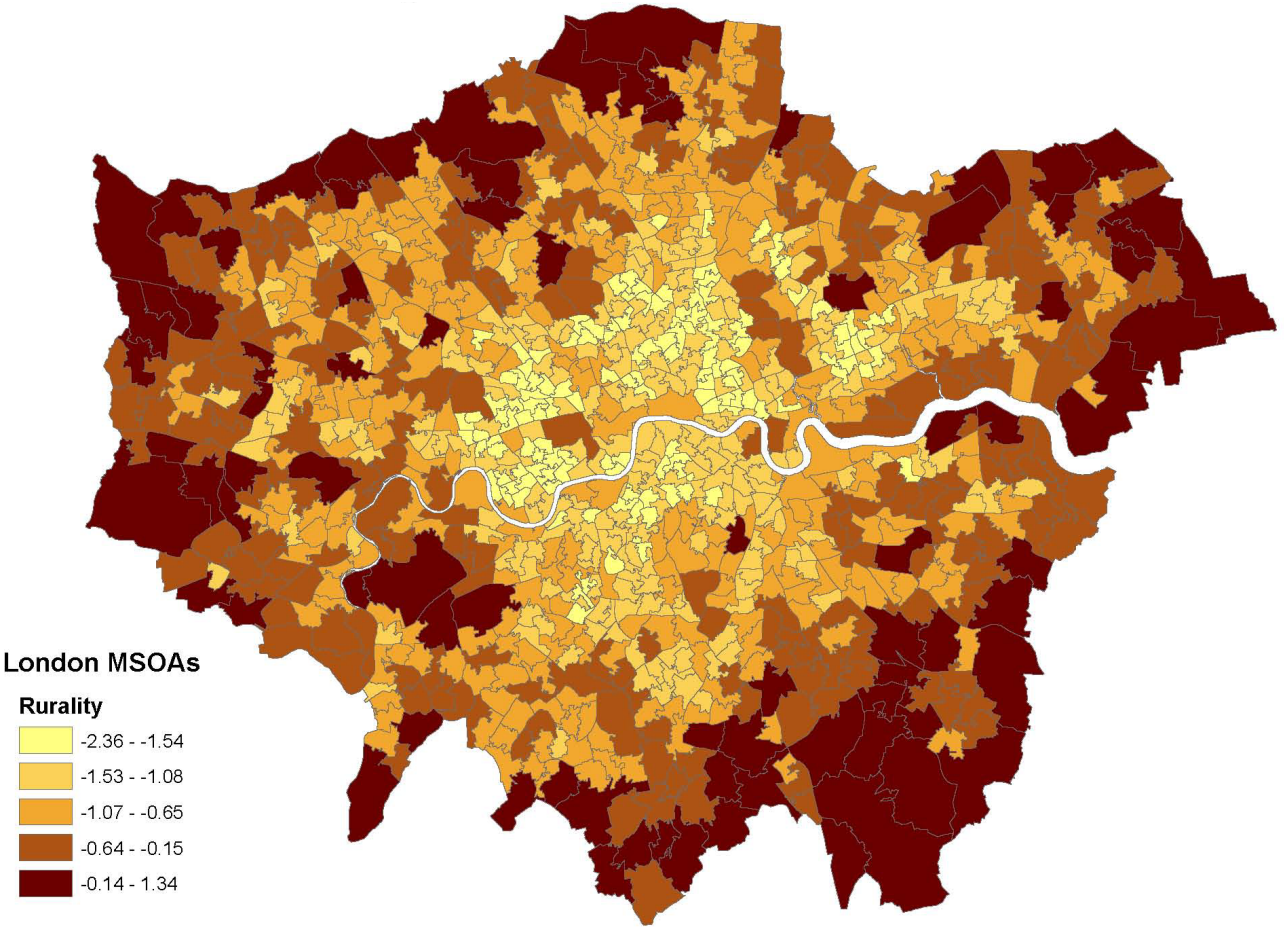
Rurality scores in London.

Here a rurality score (which amounts also to an inverse index of urbanicity) is based on % greenspace, road distance to a Post Office, road distance to a food shop, road distance to a General Practitioner, road distance to a primary school, and population density. These have the benefit of all being based on measures contemporary to the health outcomes. The access scores are included since longer drive times to key services are a distinguishing feature of rural areas, especially remote rural areas [41], and “for people living in rural areas access to services can be defined more geographically in distance to services and time taken to travel to those services” [42]. Population density has been used as an indicator of urbanity-rurality in previous suicide studies [43], while greenspace is included because of evidence that the availability of greenspace might be an important factor in explaining urban-rural health differences [44]. The leading component accounts for 68% of the original variation. Figure 1 shows a map of the resulting scores in London MSOAs. The rurality scores have a correlation of −0.47 with deprivation and −0.51 with fragmentation.

## 4. Case Study: The Data on Suicide and Self-Harm

The data on suicide available to the present case study consists of self-harm hospital stays (2006/2007 to 2010/2011) and suicide deaths (2006–2010) across MSOAs in England. The data are aggregated to areas, and are not available for individual deceased or patients. Both events are based on the area of residence (of the deceased for suicides, and of the patients for self-harms) and the hospital stays are grouped by date of discharge.

The available data on self-harm across English MSOAs and developed by the Association of Public Health Observatories for the UK Department of Health, are based on ICD10 codes X60 to X84 (intentional self-harm). The self-harm data relate only to admissions to hospital (necessarily more serious events) and do not include presentations at A&E departments where the patient is not subsequently admitted. As is customary with such data, repeat hospitalisations by the same patient are included, and this approach can be justified (e.g., terms of public health prioritisation) in terms of the higher morbidity that repeat hospital admissions imply. Repeat self-harm is an important contribution to the disease burden implied by self-harm [45]. It may be noted that self-harm events are not necessarily attempted suicides in the sense of clear suicidal intent [46], though deliberate self-harm is a strong risk factor for subsequent suicide [47,48]. Hospital admissions for self-harm (as considered here) will necessarily be capturing more serious self injuries or self-poisoning. In fact, recent official reports relating to self-harm admissions in England show that nearly 90% were caused by self-poisoning [49].

The self-harm data are only available for all persons and subject to confidentiality threshold: MSOA admission counts of 5 or under are not disclosed, lessening the potential value of further disaggregated data. It should be remembered that the ratio of self-harm events to suicides differs between genders, although available UK work with a broad geographic coverage (e.g., [50]) suggests that the impact of factors such as deprivation and social fragmentation is broadly similar as between male and female self-harm.

Suicide deaths are as defined by the UK Office of National Statistics, namely deaths given an underlying cause of intentional self-harm or an injury/poisoning of undetermined intent (ICD10 X60–X84 and Y10–Y34). Suicide deaths are available by gender. Considering all persons suicide there are 21,040 deaths over the period, with an average of 3.1 in each MSOA; 488 MSOAs (7% of all areas) had zero suicide deaths, whereas 1,461 had five or more deaths, and 80 areas had 10 or more suicide deaths.

For neither outcome are the data available by age, mainly because of data confidentiality protocols, since disaggregation both by MSOA (small areas of under 10,000 population) and by age raises issues of potential patient identifiability. Data sparsity also increases with disaggregation (with high proportion of zero counts), making regression findings less precise, and increasing the potential need for zero-inflated Poisson regression. A previous study [51] with access to disaggregated suicide data by broad age band over a seven year period showed differences between age/sex groups in spatial patterning, and there may also be some differences in the effect of area socioeconomic variables by age [52].

## 5. Case Study: Aspects of Statistical Methods

A Bayesian methodology is adopted with models estimated using the R-INLA package. Bayesian analysis reports results in terms of posterior means (analogous to classical estimates) and credible intervals (analogous to confidence intervals). Models with linear, parametric nonlinear (a cubic spline), and smooth regression effects for the area constructs, namely deprivation, social fragmentation and rurality are compared. For the cubic spline, five equally spaced inner knots are used located at the 16.6th, 33.3th, 50th, 66.7th and 83.3th percentiles, while the smooth regression effect involves a first order random walk model. Also compared are models without area effects, with spatially structured random effects only, with heterogeneity effects only, and with both spatial and heterogeneity effects.

To determine the most appropriate model, the Deviance Information Criterion or DIC is used [53], obtained as the sum of the average deviance, and a measure of complexity. The average deviance summarizes the fit, and has been used to compare models informally, but lower deviance values may be due to a high number of parameters. Hence one may penalize the average deviance by the number of parameters (measuring the complexity of the model). The number of parameters has to be estimated when the model includes random effects, and is obtained as the difference between the average deviance and the deviance at the posterior mean of the parameters. The DIC is therefore analogous to penalized measures of fit used in classical statistics such as the Akaike Information Criterion (AIC). Smaller values of the DIC suggest a better-fitting model which is at the same time parsimonious in terms of parameters used. Spiegelhalter *et al*. [53] suggest DIC differences under 2 between models are inconclusive (the model with a marginally higher DIC still deserves consideration), but that DIC differences of 3 or more favour the model with a lower DIC.

## 6. Case Study: Regression Findings

This section considers alternative regression approaches, as discussed above, to assess the impacts of the three component scores (X_1_ = deprivation, X_2_ = fragmentation, X_3_ = rurality) on suicide deaths (Section 6.1) and self-harm hospital stays (Section 6.2). Inferences regarding the joint pattern of the two outcomes are considered in Section 6.3. 

### 6.1. Regression Results for Suicide Deaths

Table 1 shows that for total suicide (for males and females combined) the lowest DIC is obtained for a linear effects model with spatial effects only, namely:

log(ρ_i_) = α + X_i1_β_1_ + X_i2_β_2_ + X_i3_β_3_ + s_i_ (3)

The difference between the DIC for this model (26,488.5) and the closest competitor (26,489.1, for a model including both spatial and heterogeneity effects) is admittedly small, but a preference for this model can be justified in wider parsimony terms, that a model with better fit and fewer parameters is to be preferred. The worse fitting model in this comparison has nearly 7,000 extra parameters (the h_i_ terms), and in fact none of these effects is significant in terms of 90% credible intervals excluding zero. By contrast, over 600 of the spatial effects s_i_ in this model have significant 90% credible intervals. The regression effects are anyway virtually identical between the two models.

**Table 1:** DIC According to Regression Type and Area Effects, Suicide Deaths.

Form of Regression Effects	Area Effects	Deviance Information Criterion
Linear	None	26,933.9
	Heterogeneity	26,762.1
	Spatial & Heterogeneity	26,489.1
	Spatial only	26,488.5
Smooth Regression	None	26,912.0
	Heterogeneity	26,753.1
	Spatial & Heterogeneity	26,506.8
	Spatial only	26,506.9
Cubic Spline	None	26,898.2
	Heterogeneity	26,740.6
	Spatial & Heterogeneity	26,498.0
	Spatial only	26,497.0

Despite this possible uncertainty about the best fitting model, it is clear that area effects are necessary, and that a model with spatially correlated area effects provides a suitable fit and hence a suitable representation of unobserved small area influences on suicide. Table 2 shows the regression coefficients (β_1_ to β_3_), together with implied relative suicide risks at extreme component scores (5th and 95th percentiles). The scores are in standardized form (mean zero and standard deviation one), so that the size of regression coefficients is a direct measure of their impact on the outcome.

**Table 2:** Regression coefficients and predicted relative risks, suicide deaths.

Impacts (parameters) on log of Relative Suicide Risk			
	Mean	2.5%	97.5%
Deprivation	0.157	0.137	0.177
Fragmentation	0.134	0.115	0.152
Rurality	0.041	0.020	0.061
**Predicted Suicide Relative Risk at Extreme Construct Scores**			
	**5th percentile**	**95th percentile**	**Ratio**
Deprivation	0.82	1.36	1.65
Fragmentation	0.85	1.29	1.52
Rurality	0.95	1.09	1.15

The impact of fragmentation as identified in more localized studies [27,28] is here confirmed at a national scale across English small areas. Thus the 50 MSOAs across England with the highest social fragmentation scores have a total of 313 suicides during 2006–2010, as against 192.5 expected on the basis of England wide rates, an SMR of 163. This group of MSOAs includes individual areas with unusually high numbers of suicide deaths, such as Leicester 024 (24 deaths against five expected), Bournemouth 019 (13 deaths against 3.6 expected) and Plymouth 029 (with 16 deaths against 4.9 expected).

An additional feature is the positive impact of rurality (β_3_ = 0.041) after allowing for deprivation and fragmentation. The zero order correlation between rurality and raw suicide SMRs is unreliable as a measure of association because of the instability of SMRs based on small numbers of suicide deaths in many MSOAs [54,55]. Instead to gauge the unmediated impact of rurality without control for the other two constructs, a reduced regression is applied, namely:

log(ρ_i_) = α + X_i3_β + s_i_ (4)

containing only rurality and a spatially structured area effect. This regression shows a negative impact of rurality (*i.e.*, higher suicide risks in urban areas), with a central estimate for β (and 95% credible interval) of −0.10 (−0.12, −0.08). The fact that rurality becomes a positive risk factor after allowing for other urban dimensions is in agreement with studies suggesting an attenuation of the impact on suicide of high levels of urbanicity. Thus [17] find that the “bull’s-eye” pattern of increases in suicide rates from the suburbs to the centre of London apparent in 1981–1985 is no longer present in 2001–2005.

**Figure 2:**
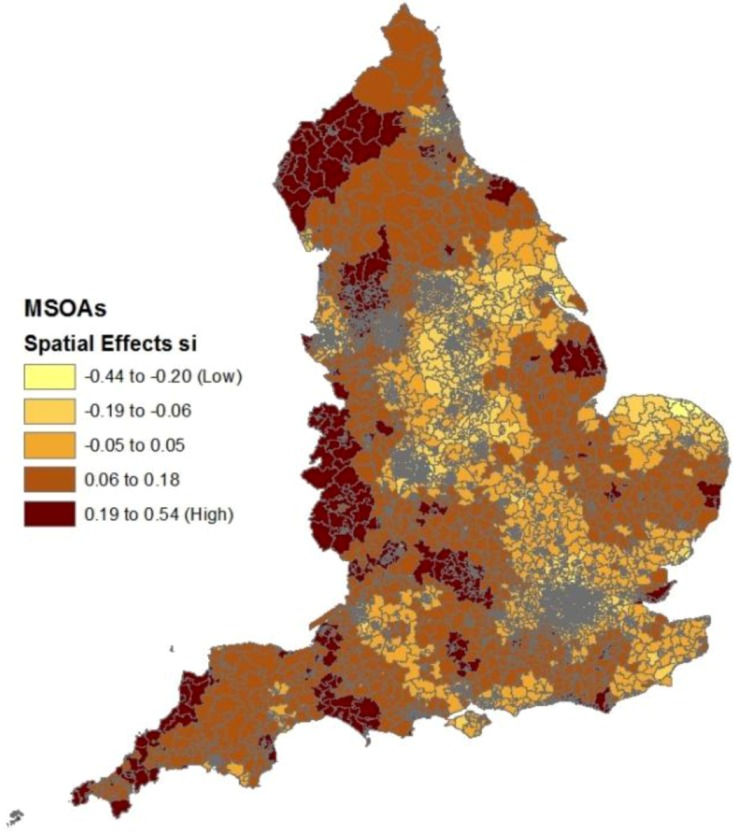
Spatial effects in suicide regression after controlling for construct scores.

Figure 2 shows clusters of small areas with relatively high spatial effects (s_i_ parameters), indicating spatial clustering of risk factors apart from deprivation, fragmentation and rurality. These include clusters in the extreme North West (especially the county of Cumbria), the extreme South West (Cornwall) and parts of the West Midlands adjacent to the Welsh border (Hereford, Shropshire).

Table 3 shows a similar finding to Table 1 for suicide deaths by gender, namely that a linear effects model has the lowest DIC as compared to the two nonlinear approaches. 

**Table 3:** DIC According to regression type and area effects, suicide deaths by gender.

	Form of Regression Effects	Area Effects	DIC
Males	Linear	None	24,651.5
		Heterogeneity	24,519.2
		Spatial & Heterogeneity	24,355.6
		Spatial only	24,361.3
	Smooth Regression	None	24,636.3
		Heterogeneity	24,514.3
		Spatial & Heterogeneity	24,370.2
		Spatial only	24,377.0
	Cubic Spline	None	24,623.8
		Heterogeneity	24,503.5
		Spatial & Heterogeneity	24,365.0
		Spatial only	24,370.6
Females	Linear	None	15,315.2
		Heterogeneity	15,314.6
		Spatial & Heterogeneity	15,239.6
		Spatial only	15,216.0
	Smooth Regression	None	15,312.6
		Heterogeneity	15,312.6
		Spatial & Heterogeneity	15,217.8
		Spatial only	15,217.6
	Cubic Spline	None	15,325.2
		Heterogeneity	15,325.1
		Spatial & Heterogeneity	15,232.8
		Spatial only	15,231.8

For females, the best model combines linear predictor effects with spatial area effects, but for males, a lower DIC emerges for linear effects combined with both spatial and unstructured area effects. For males the DIC differences for this model against other models are relatively large, but for females the difference against the best completing model is smaller at 1.6, though a better fitting model which is additionally simpler is arguably preferable to focus on. Table 4 shows that for males, all three area constructs are significant positive suicide risk factors. Deprivation is the strongest influence on male suicide mortality, but fragmentation is a significant secondary influence. For females by contrast, fragmentation is the leading ecological risk factor, and the other constructs have weak (albeit positive) effects. To illustrate the fragmentation effect, there are 77 female deaths through suicide in the 50 MSOAs with the highest fragmentation scores, as against 39.7 expected, an SMR of 194. Effect modification by gender can be assessed by comparing the 95% credible intervals for the three predictors, and it is apparent that deprivation and fragmentation effects are distinct between genders (fragmentation a stronger influence on female suicide, deprivation a stronger influence on male suicide), but that rurality effects do overlap. 

**Table 4:** Regression coefficients and predicted relative risks, suicide deaths by gender.

Impacts (parameters) on log of Relative Suicide Risk						
	Males			Females		
	Mean	2.5%	97.5%	Mean	2.5%	97.5%
Deprivation	0.191	0.169	0.214	0.025	-0.013	0.063
Fragmentation	0.121	0.100	0.141	0.179	0.145	0.212
Rurality	0.051	0.028	0.075	0.026	-0.013	0.065
**Predicted Suicide Relative Risk at Extreme Construct Scores**						
	**Males**			**Females**		
	**5th percentile**	**95th percentile**	**Ratio**	**5th percentile**	**95th percentile**	**Ratio**
Deprivation	0.788	1.448	1.837	0.969	1.050	1.083
Fragmentation	0.862	1.257	1.458	0.803	1.403	1.747
Rurality	0.937	1.110	1.185	0.967	1.055	1.091

### 6.2. Regression Analysis for Self-Harm

Whereas linear effects are suitable to describe the effects of the three constructs on suicide deaths, Table 5 shows that the two nonlinear approaches have lower DICs, and furthermore that unstructured effects are needed to account for residual overdispersion even after allowing for structured area effects. 

**Table 5:** DIC according to regression type and area effects, self-harm Data.

Regression Effects	Area Effects	DIC
Linear	None	158,084
	Heterogeneity	53,079
	Spatial & Heterogeneity	52,043
	Spatial only	52,143
Smooth Regression	None	139,365
	Heterogeneity	53,006
	Spatial & Heterogeneity	51,975
	Spatial only	52,124
Cubic Spline	None	139,532
	Heterogeneity	52,967
	Spatial & Heterogeneity	51,940
	Spatial only	52,090

**Figure 3:**
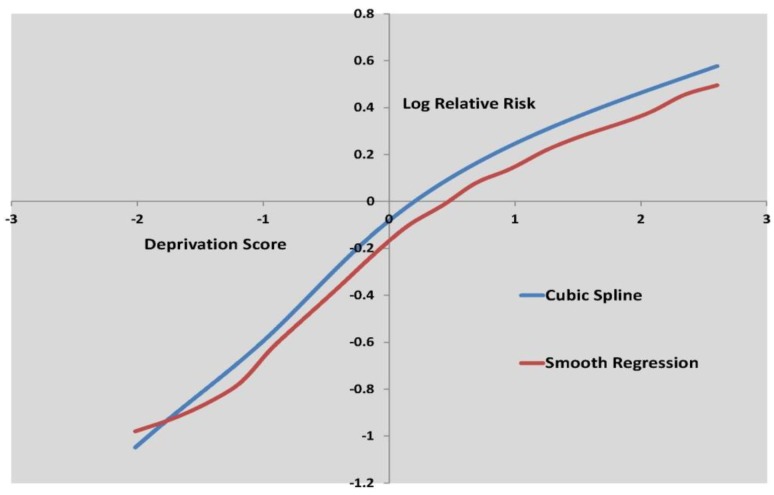
Log relative risk by deprivation score, cubic spline and smooth regression.

Figure 3 to Figure 5 show the log relative risk of self-harm as the scores vary over their range, according to the two best fitting nonlinear models.

**Figure 4:**
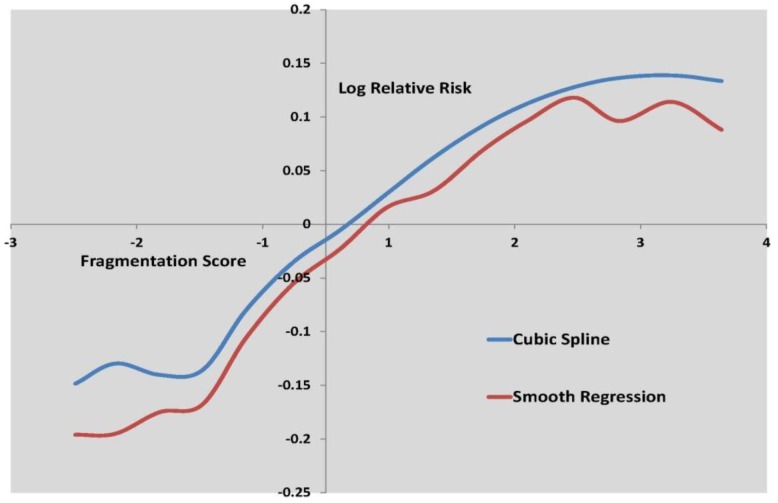
Log relative risk by fragmentation score, cubic spline and smooth regression.

**Figure 5:**
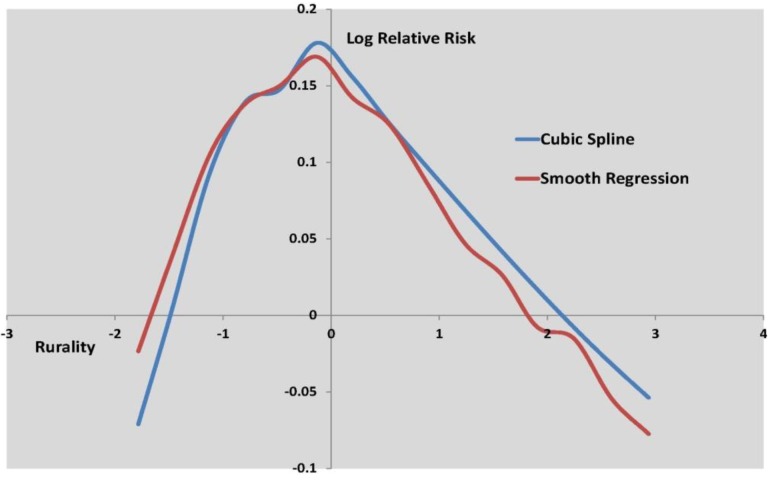
Log relative risk by rurality score, cubic spline and smooth regresssion.

Figure 3 shows curvature in the deprivation effect, while Figure 4 shows deceleration in increased self-harm risk at higher fragmentation levels. Table 6 shows that deprivation is by far the stronger influence on small area self-harm variations, with a risk ratio of 3.19 for areas with high deprivation scores (95th percentile) as against low deprivation scores (5th percentile). 

**Figure 6:**
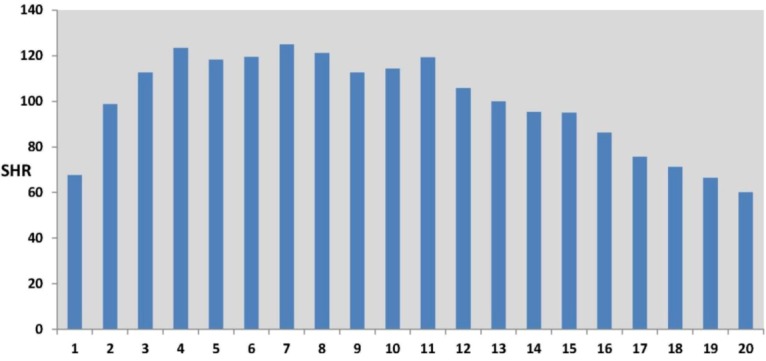
Self-Harm Standard Hospitalisation Ratios (SHRs) by 5-Percentile Rurality Categories (Category 1, lowest rurality, to Category 20, highest rurality).

Figure 5 shows a more marked nonlinearity in the effect of rurality on self-harm. Lower than average self-harm rates characterize areas with the lowest and highest rurality scores, where the lowest rurality scores are typically in inner and central city areas. The highest self-harm rates occur in areas with intermediate rurality scores, typically suburban areas. This effect is also apparent in the original data (see Figure 6), namely in the profile of observed SHRs (standard hospitalization ratios) over twenty percentile categories of rurality (category 1 defined by the 5% of MSOAs with the lowest rurality scores, category 2 by the next lowest 5% of scores, etc, up to category 20 containing the 5% of MSOAs with the highest rurality scores). 

**Table 6:** Relative risks of self harm at selected percentile points of constructs, under cubic spline regression model.

Percentile	Deprivation		Fragmentation		Rurality	
	Relative Risk at Percentile	Ratio to 5th percentile	Relative Risk at Percentile	Ratio to 5th percentile	Relative Risk at Percentile	Ratio to 5th percentile
5th	0.575	1.000	0.887	1.000	0.953	1.000
25th	0.733	1.275	0.931	1.049	1.039	1.090
50th	0.973	1.693	0.991	1.117	1.076	1.129
75th	1.332	2.317	1.053	1.187	1.035	1.087
95th	1.834	3.192	1.160	1.307	0.907	0.952

In particular, self-harm rates may be elevated in relatively deprived suburban estates consisting of social (public sector rented) housing, sometimes denoted as “suburban social housing” [56]. Of the 100 MSOAs with the highest self-harm rates (with a collective self-harm SHR of 361, or 3.6 more self-harm hospital stays than average), 54 are in categories 4 to 8 of the twenty rurality percentile categories, as compared to 24 in categories 1 to 3 (lowest rurality). They have on average 39.2% of households in social rented housing (2001 Census), compared to an average of 19% social rented housing across all 6,781 MSOAs.

The above illustrated nonlinear effect is concealed by the linear regression (with spatial and unstructured area effects included) which has a clearly higher DIC of 52,043, compared to the nonlinear alternatives (51,975 for smooth regression, and 51,940 for spline regression). 

**Figure 7:**
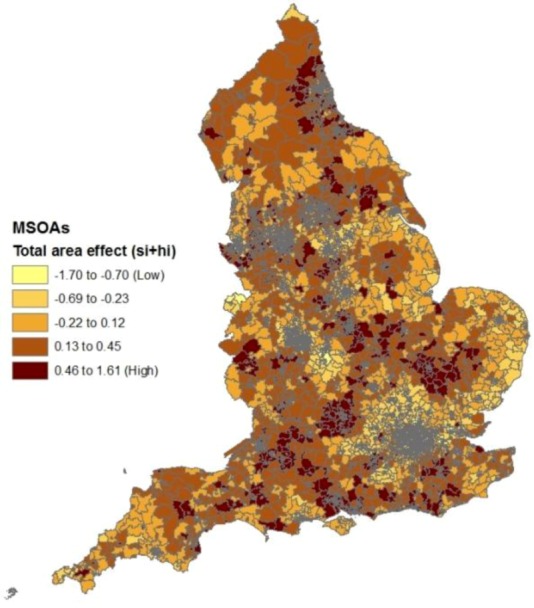
Area effects in self-harm regression after controlling for construct scores.

The linear regression model gives a negative rurality effect β_3_, and would predict highest self-harm rates in central city areas. The linear regression option provides estimated coefficients (and 95% intervals) of β_1_ = 0.347 (0.335, 0.359) for deprivation, β_2_ = 0.078 (0.066, 0.089) for fragmentation, and β_3_ = −0.081 (−0.092, −0.069) for rurality. A simple urban-rural binary split would also not detect this form of nonlinear effect.

A referee has pointed out that only a minority of self-harm attendances at A&E units are admitted to hospital (e.g., [57]), and the nonlinear association may partly reflect that people from rural areas (with lesser accessibility to hospitals) are less likely to attend hospital, and those that do may be less likely to be admitted.

Figure 7 shows the spatial pattern of the composite area effects (unexplained by deprivation and the two other constructs), namely t_i_ = s_i_ + h_i_. These show clear differences to the spatial effects from the suicide regression (in Figure 2), with no apparent unexplained excess risk in Cumbria and Cornwall. On the other hand, the correlation between the two sets of effects (t_i_ in Figure 7 and s_i_ in Figure 2) is 0.50, suggesting common unobserved risk factors for the two outcomes.

### 6.3. Interrelationships between the Outcomes

The overall modeled risk for each event (suicide, self-harm) is based on combining the impacts of the regression (on deprivation, *etc.*) and of the unobserved area effects. To some degree, high risks for the two events occur in distinct area types. Areas with the highest suicide risk include areas with high population turnover and often extensive private sector renting; in England, such areas are in central areas in large cities, as well as university and coastal towns, which often have mobile sub-populations, as recognized in parliamentary discussion [58]. Areas with the highest self-harm risk include deprived inner and outer city areas of social rented (public sector) housing. 

Nevertheless, a considerable degree of overlap in the pattern of the two events is also apparent. There is a relatively high correlation of 0.64 between fitted relative risks for suicide (persons) and self-harm, and overlap also shows when fitted relative risks are grouped into deciles (Table 7).

**Table 7:** Overlap in relative risk deciles (deciles of fitted relative risks, best fitting models).

Self-Harm											
Suicide	Decile 1	Decile 2	Decile 3	Decile 4	Decile 5	Decile 6	Decile 7	Decile 8	Decile 9	Decile 10	Total
Decile 1	241	139	99	80	54	33	15	11	6	0	678
Decile 2	144	102	108	93	85	73	39	24	8	2	678
Decile 3	75	112	94	97	98	76	56	42	23	5	678
Decile 4	68	88	104	83	82	74	86	56	25	12	678
Decile 5	51	75	85	81	74	96	85	62	51	18	678
Decile 6	39	59	54	73	80	113	90	76	65	29	678
Decile 7	21	42	50	68	82	80	85	105	100	45	678
Decile 8	21	34	39	50	59	63	94	106	127	85	678
Decile 9	15	21	38	34	42	44	76	122	139	147	678
Decile 10	3	6	7	19	22	26	52	74	134	336	679
Total	678	678	678	678	678	678	678	678	678	679	6,781

Thus 336 of the 679 areas in the top decile of self-harm relative risk are simultaneously in the top decile of suicide relative risk. The ratio of self-harm rates to suicide rates is also a potential area for research: the England wide rates for the two events are 10.1 per 100,000 (suicide) and 190 per 100,000 (self-harm), but at MSOA level the ratio of self-harm rates to suicide rates shows an asymmetric positive skew pattern, with some 200 MSOAs showing self-harm to suicide ratios exceeding 38 (twice the England ratio).

## 7. Discussion

This paper has considered data on both suicide deaths and self-harm hospital stays across 6,781 small areas in England. This comprehensive national coverage (of one of the UK constituent countries) provides scope for assessing findings from smaller scale city or regional studies. Additionally the methodology used has assessed the most suitable form of regression effect (linear or nonlinear), rather than assuming linearity by default, and also allowed for the impact of spatially clustered unobserved factors. Findings reported above show how construction of predictors, for example using a continuous score rather than a two or three fold categorization, can be important in detecting nonlinearity. While the linearity assumption is satisfactory for suicide mortality, for self-harm there is nonlinearity in the effects of area socioeconomic predictors.

Some small area studies of suicide mortality have been national in coverage, though previous studies of small area self-harm variations are fewer and often relatively localized in terms of geographic coverage. However, national coverage (across Ireland) is obtained in an ecological study [22] of self-harm variation which shows area deprivation as the primary influence. The primacy of deprivation as an ecological risk factor for self-harm is confirmed here in a study across English small areas.

By contrast, the present study demonstrates a more multifactorial ecological risk profile for suicide mortality, with deprivation, fragmentation and rurality all being positive risk factors for total suicide mortality. When male and female suicide are compared, fragmentation has a stronger influence on female suicide [28].

The positive effect of both deprivation and fragmentation on suicide for persons and males confirms earlier UK studies (e.g., [27,28]) with more restricted geographic coverage. It is possible that in fact the impact of fragmentation may be attenuated to some degree, since in constructing the fragmentation score, data for some indicators is based on the 2001 Census, some five years earlier than the suicide data (this factor may also weaken the association of fragmentation with self-harm rates).

The positive effect of rurality on suicide deaths, after allowing for the other two constructs, confirms the England and Wales study of Middleton *et al*. [43] which was confined to young adults and concerned with the pre-2000 period. In other countries, such as the US and Australia, rurality is also a suicide risk factor [33,59].

The geographic location of areas with high suicide risk and high self-harm risk, and of overlapping risk in both outcomes (see Section 6.3), shows interesting aspects that might be the focus of further research, for example to explain spatial clustering in unobserved area risk factors, or accumulate evidence for suicidogenic area contexts. An improved explanation in regression terms for both types of outcome possibly rests with the introduction of variables representing special area types, such as coastal towns [17,58] or outer city social housing areas.
